# Quand le bloc axillaire reste la seule alternative chez un enfant de 5 ans

**DOI:** 10.11604/pamj.2015.21.36.5343

**Published:** 2015-05-18

**Authors:** Mohamed Said Nakhli, Nawel Béjaoui, Walid Naija, Salah Mhamdi, Rachid Said

**Affiliations:** 1 Service d'Anesthésie Réanimation, CHU Sahloul, Sousse, Tunisie

**Keywords:** Anesthésie pédiatrique, bloc axillaire, hépatite aigue, kyste hydatique pulmonaire, pediatric Anesthesia, axillary block, acute hepatitis, Pulmonary hydatid cyst

## Abstract

Le bloc axillaire par neurostimulation chez l'enfant n'est pas une technique habituelle, surtout avec le développement des techniques d'anesthésie locorégionale échoguidées. Cependant elle peut être une alternative intéressante si l'anesthésie générale est a risque voir même contre indiquée. Nous rapportons le cas d'un bloc axillaire anesthésique chez un enfant de 5 ans porteur d'un kyste hydatique du poumon et une hépatite A sévère proposé pour une fixation de fracture du condyle externe de l'humérus.

## Introduction

La particularité de la population pédiatrique rend difficile la pratique des blocs plexiques contrairement aux adultes. Les difficultés techniques, le risque de complications, la faible expérience des équipes et la rareté des références bibliographiques a rendu peu fréquente la réalisation de ces blocs chez l'enfant. Cependant, la plupart des techniques d'anesthésie locorégionale dans la population pédiatrique se fait chez des enfants sous anesthésie générale. Nous rapportons le cas d'un bloc axillaire anesthésique chez un enfant de 5 ans porteur d'un kyste hydatique du poumon et une hépatite A sévère proposé pour une fixation de fracture du condyle externe de l'humérus droit.

## Patient et observation

L'enfant W.K âgé de 5 ans, ayant un poids de 20 Kg, sans antécédents, admis au service d'orthopédie pour fracture du condyle externe de l'humérus droit suite à un accident de loisir. L'intervention jugée semi urgente, une fixation par embrochage a été proposée par l’équipe d'orthopédie.

L’évaluation pré-anesthésique trouve un ictère conjonctival avec notion de douleurs abdominales évoluant depuis une semaine. L’état général était conservé. Le bilan hépatique est revenu très perturbé: ASAT à 564UI /l (15 fois la normale), ALAT à 962 UI /l (24 fois la normale), bilirubine totale à 134 UI/l (8 fois la normale), bilirubine conjuguée à 84UI/l (12 fois la normale) et un taux de prothrombine à 36%.

Les sérologies d'hépatite virale pratiquées selon la technique d'Electro-chemiluminescence Immunoassay (ECLIA) automatisée, (Cobas e411, Roche^®^) ont conclu à une hépatite A aigue (Anti HAV IgM positifs).

Une échographie abdominale faite est revenue normale. La radiographie de thorax demandée dans le cadre du bilan pré opératoire a montré une image kystique hyperdense du poumon droit pouvant cadrer avec un kyste hydatique pulmonaire. Ce diagnostic a été confirmé par une TDM thoracique en montrant un kyste hydatique du lobe supérieur droit non compliqué. Par ailleurs, l'enfant ne présentait aucune symptomatologie respiratoire.

Etant donné que ce type de fracture chez l'enfant doit être rapidement fixé, un délai de 10 jours pour l'amélioration du bilan hépatique ne peut être attendu. De même le kyste hydatique découvert fortuitement présentait un risque réel de fissuration ou de rupture lors de la ventilation mécanique durant l'anesthésie. Le challenge pour cet enfant était de réaliser l'ostéosynthèse du coude, tout en évitant une anesthésie générale avec ventilation mécanique et utilisation de produits anesthésiques qui pourraient aggraver la fonction hépatique.

Nous avons alors décidé de réaliser une anesthésie locorégionale par un bloc axillaire par neurostimulation moyennant une légère sédation par rémifentanil en perfusion. Un consentement oral des parents a été obtenu, après leur avoir expliquer les modalités et les risques de cette technique.

L'enfant a été installé en position de décubitus dorsal, et un abord vasculaire périphérique a été mis sur le dos de la main gauche. Une analgésie a été réalisé par une perfusion continue de remifentanil à la dose de 0.2 µg/kg /min associée à des bolus de 10 mg de propofol à la demande. L'enfant est resté en ventilation spontanée sous lunette d'oxygène à 3 l/min, facilement réveillable (Richmond Agitation Sedation Scale à -2).

Le membre supérieur droit a été installé en abduction à 90°. Après désinfection chirurgicale de la région axillaire nous avons procédé au blocage nerveux par la technique de multi stimulation avec une aiguille de 24G, 25 mm (Vygon, France). L'anesthésique local utilisé était la lidocaïne adrénalinée à 1% à raison de 3 ml par nerf. L'intensité électrique minimale requise était de 0,5 mA. Les nerfs stimulés étaient bloqués selon l'ordre suivant: le nerf musculo-cutané, le nerf médian, le nerf radial enfin le nerf cubital. Un complément du nerf cutané médial du bras et de l'avant bras a été réalisé par infiltration à la lidocaïne à 1%.

Cinq minutes après, un bloc sensitif et moteur s'est installé sans incidents. La perfusion de rémifentanil a été poursuivie au cours de l'acte avec des doses entre 0,05 et 0,2µg /kg /min garantissant une sédation légère et évitant les mouvements de l'enfant. L'acte chirurgical consistait en un embrochage percutané du coude sous garrot pneumatique placé à la racine du membre supérieur droit. L'intervention a duré 30 min au cours desquels l'enfant est resté en ventilation spontanée sans aucune modification des paramètres vitaux.

Une analgésie post opératoire a été anticipée par Ibuprofène sirop après le réveil de l'enfant et avant la levée du bloc sensitif, et les antalgiques à base de paracétamol ont été proscrit. Aucune complication neurologique secondaire au bloc axillaire n'a été constatée à 24 et 48 heures post opératoire. Le bilan hépatique s'est amélioré progressivement les jours suivants.

## Discussion

L′anesthésie locorégionale semble devenir la pierre angulaire dans la pratique de l′anesthésie pédiatrique. On y voit souvent l′association anesthésie générale et locorégionale afin d′assurer une épargne morphinique, une atténuation du stress chirurgical, une analgésie post opératoire de qualité ainsi qu′une réhabilitation post opératoire rapide [1].

Mais souvent réaliser un bloc plexique avec neurostimulation chez l′enfant de bas âge peut devenir un challenge pour le médecin anesthésiste à cause de la rareté des repères anatomiques et de la variabilité de la profondeur des nerfs.

L′approche axillaire du plexus brachial chez l′enfant remonte aux années soixante [2]. Plusieurs variantes de cette approche ont été décrites, mais en raison de sa faible morbidité, elle reste la plus utilisée par rapport aux autres blocs du membre supérieur [3]. Cependant, elle est souvent associée à un inconfort lié à la position douloureuse en abduction à 90° du membre supérieur. Cela requiert une sédation profonde voire même une anesthésie générale, avec un risque non négligeable de lésions nerveuses et de toxicité systémique masquée par l′anesthésie.

Chez l′enfant et contrairement à l′adulte, la diffusion des anesthésiques locaux est plus facile à travers la gaine neuro vasculaire au niveau axillaire, car les septas séparant les nerfs médians, radiaux et cubitaux sont très fines. Donc la technique d′injection unique semble aussi efficace que la technique à injections multiples [2].

La pratique de l′anesthésie locorégionale doit obéir à un ensemble de règles de sécurités [4] car la toxicité des anesthésiques locaux est un autre problème en anesthésie pédiatrique. Il a fallut attendre les années 90 pour que des recommandations sur la sécurité en anesthésie locorégionale pédiatrique voient le jour. Les produits habituellement utilisés sont les anesthésiques locaux du groupe amino amides [4].

Il est admis chez l′enfant que la dose maximale de lidocaïne sans vasoconstricteur est de 5mg/kg par bloc, alors qu′elle de 7 mg/kg pour la lidocaïne adrénalinée [5]. Dans notre cas la dose totale utilisée était de 120 mg de lidocaïne adrénalinée soit 5 mg/kg. La réduction des doses avait pour objectif de minimiser les effets toxiques de la lidocaïne chez un enfant avec une clairance hépatique basse secondaire à une hépatite aiguë sévère (taux de prothrombine < 50%) [6].

Chez l′enfant les critères de neurostimulation sont les mêmes que chez l′adulte. Il ne faut pas rechercher une réponse pour une intensité de stimulation < 0,5 mA [4].

Dans la littérature, on a pu retrouver un cas similaire au notre, celui d′un enfant de 17 mois qui avait une fracture du condyle externe de l′humérus et qui présentait en même temps un tableau d′hépatite aiguë médicamenteuse. La chirurgie a été réalisée sous anesthésie locorégionale par bloc inter scalénique échoguidé associé à une neurostimulation. Une sédation par fentanyl et thiopental et une ventilation au masque facial ont été nécessaire. L′anesthésique local utilisé était la ropivacaïne à 0,5% (1,5 ml) [1].

Dans notre cas, la ventilation en pression positive était proscrite, car le risque majeur était une fissuration voire même une rupture du kyste hydatique du poumon, l′enfant est resté en ventilation spontanée légèrement sédaté par remifentanil.

Avec l'avènement des techniques d'anesthésie locorégionale écho-guidées chez l'adulte, la population pédiatrique peut en tirer un grand bénéfice, et permettre ainsi son développement en tant que technique d'anesthésie et d'analgésie post opératoire.

## Conclusion

L'anesthésie locorégionale pédiatrique, semble pour certains médecins anesthésistes peu réalisable, vue la difficulté technique, les modifications anatomiques, et le taux d’échec non négligeable surtout par neurostimulation. Mais actuellement et avec le développement des techniques d'anesthésie locorégionale sous échographie, les risques et les complications ont nettement régressé. Ce cas clinique illustre la nécessité de maitriser cette technique peu habituelle dans notre pratique quotidienne pour faire face à des situations complexes, pour lesquels l'anesthésie locorégionale reste l'ultime solution.
